# Supplementary material for: LSD1 inhibition induces differentiation and cell death in Merkel cell carcinoma
Source: EMBO Mol Med. 2020 Oct 7;12(11):e12525. doi: 10.15252/emmm.202012525 (PMC7645387; doi:10.15252/emmm.202012525)
Supplement: Supplementary file 1 — Appendix [file EMMM-12-e12525-s001.pdf]

Appendix - Table of Content

Appendix Figure S1. LSD1 inhibition induces cell cycle arrest and cell death.

Appendix Figure S2. Merkel cell FACS sorting gating strategy for SMART-seq2 sequencing.

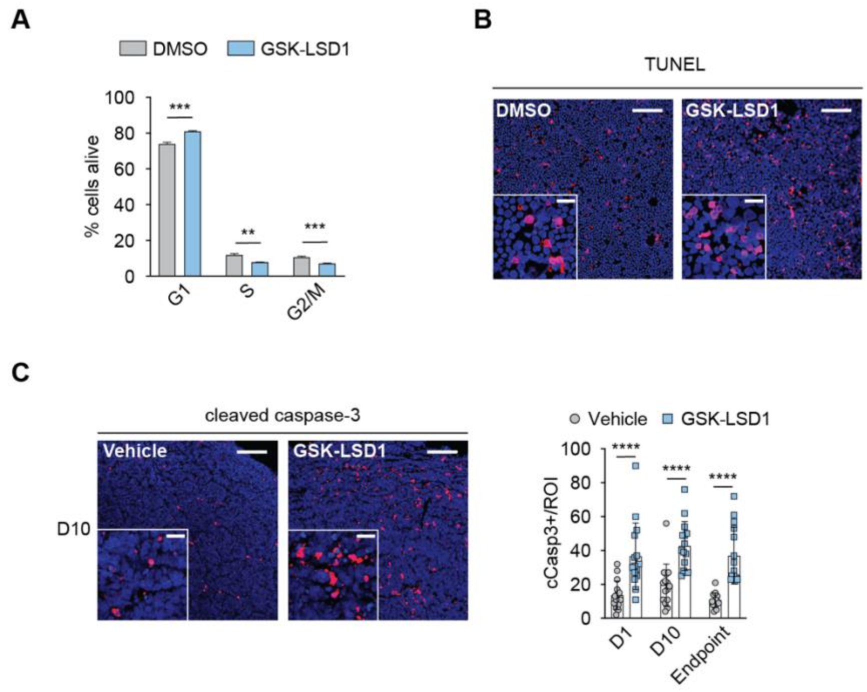

Appendix Figure S1. LSD1 inhibition induces cell cycle arrest and cell death.
A. Quantification of EdU/Propidium iodide staining after 3 days of 100 nM GSK-LSD1 or DMSO treatment. n = 3 biological replicates. Data are represented as means ±SD. **p<0.01; ***p<0.001 (G1 phase: DMSO vs GSK-LSD1 p = 0.0003; S phase: DMSO vs GSK-LSD1 p = 0.0011; G2/M phase: DMSO vs GSK-LSD1 p = 0.0004; unpaired Student's t-test).
B. Representative images of *in vitro* immunofluorescent TUNEL staining of PeTa cells after 6 days of 100 nM GSK-LSD1 or vehicle treatment from data in Fig 3J. Upper right scale bar represents 100 µm, insert scale bar represents 20 µm.
C. Left. Representative images of immunofluorescent cleaved caspase-3 staining of tumor slides after 10 days of *in vivo* GSK-LSD1 or vehicle treatment. Upper right scale bar represents 100 µm, insert scale bar represents 20 µm. Right. Quantification of cleaved caspase-3 signal of tumor slides from mice treated with 100 nM GSK-LSD1 or DMSO for 1 day, 10 days or until experiment endpoint. n = 15, ****p<0.0001 (D1 vehicle vs D1 GSK-LSD1 p<0.0001, Mann-Whitney test; D10 vehicle vs D10 GSK-LSD1 p<0.0001, Mann-Whitney test; Endpoint vehicle vs Endpoint GSK-LSD1 p<0.0001, unpaired Student's t-test with Welch's correction). ROI, region of interest.

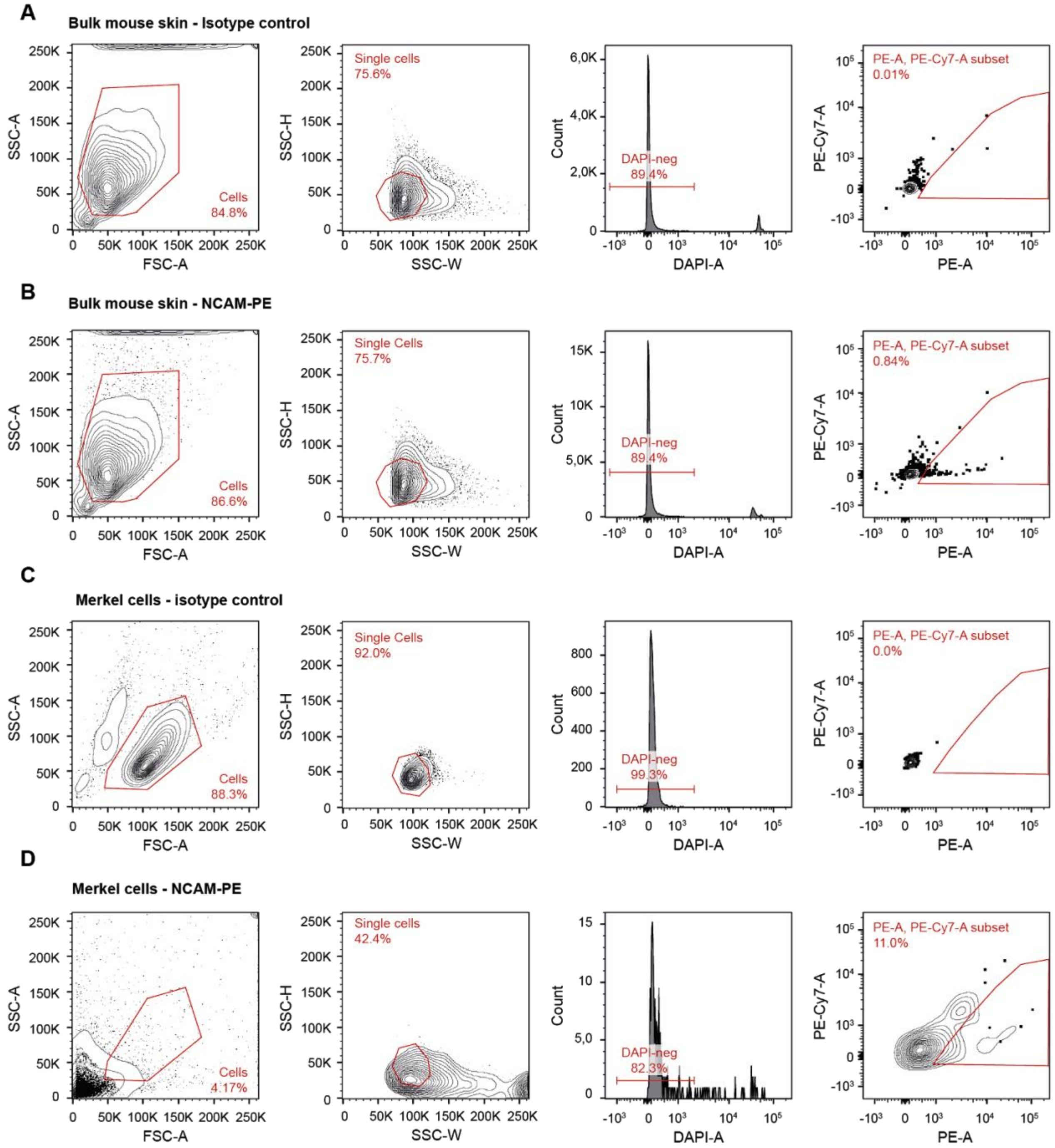

Appendix Figure S2. Merkel cell FACS sorting gating strategy for SMART-seq2 sequencing.

A. Bulk mouse skin labeled with IgM-PE mouse isotype control.

B. Bulk mouse skin labeled with NCAM-PE. DAPI- cells were sorted.

C. Merkel cells labeled with IgM-PE mouse isotype control.

D. Merkel cells labeled with NCAM-PE. DAPI-/NCAM+ cells were sorted. Each plot shows the subpopulation gated for in the preceding plot to the left.
