# Supplementary material for: LSD1 inhibition induces differentiation and cell death in Merkel cell carcinoma
Source: EMBO Mol Med. 2020 Oct 7;12(11):e12525. doi: 10.15252/emmm.202012525 (PMC7645387; doi:10.15252/emmm.202012525)
Supplement: Supplementary file 2 — Expanded View Figures PDF [file EMMM-12-e12525-s002.pdf]

Expanded View Figures

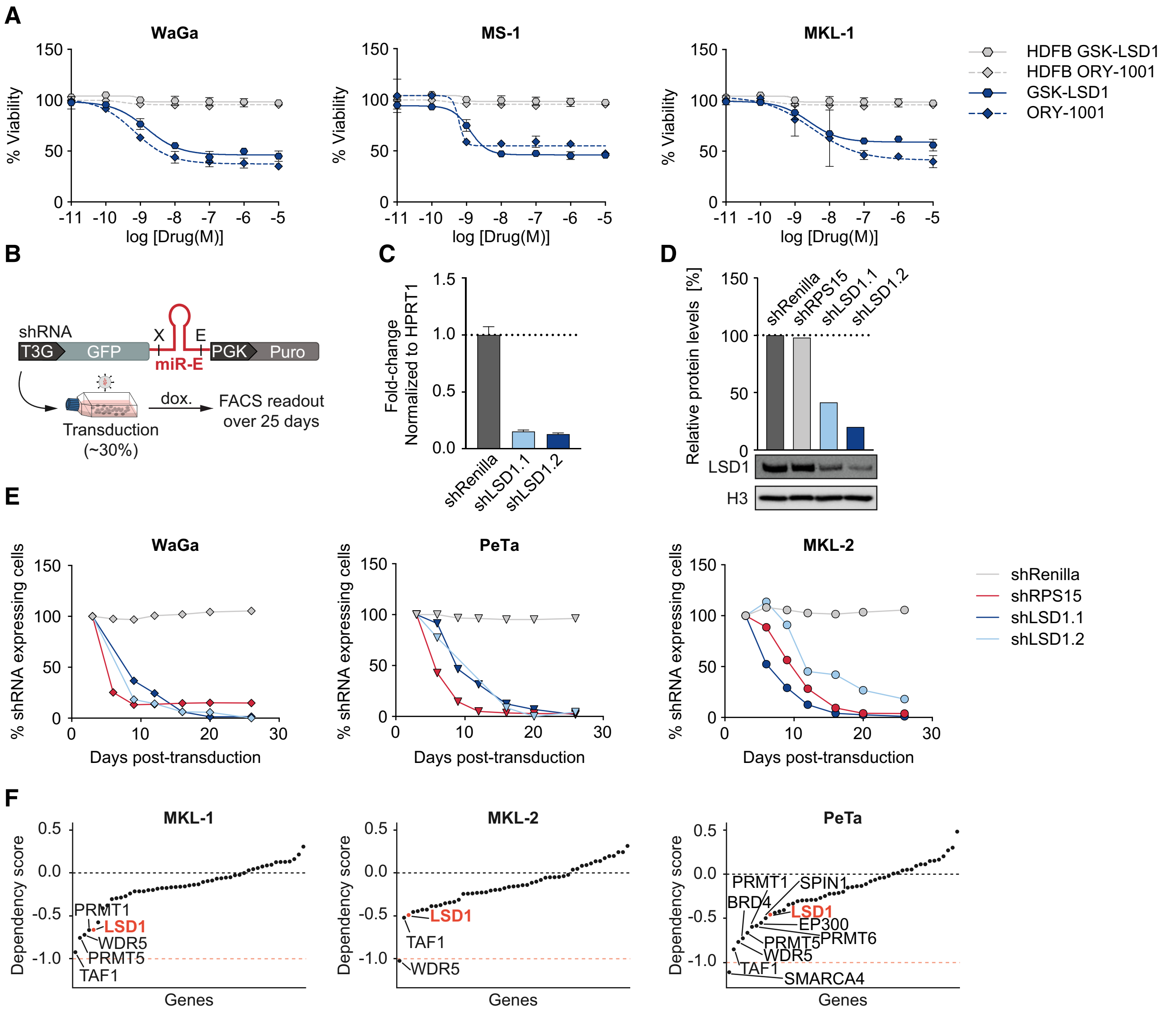

Figure EV1. LSD1 is required for Merkel cell carcinoma proliferation.

A Dose–response curves for viability of three MCC cell lines (WaGa, MS-1, and MKL-1) and control HDFB cells after 6 days of treatment with GSK-LSD1 or ORY-1001. $n = 4$ technical replicates for each sample. Data are represented as means ± SD.

B Schematic depicting the *in vitro* shRNA-based competition assay. X, XhoI restriction site; E, EcoRI restriction site; T3G, Tet-On 3G; dox., doxycycline.

C RT–qPCR of LSD1 RNA in the indicated shRNA-knockdown MKL-2 cells. $n = 4$ technical replicates. Data are normalized to housekeeping gene HPRT1 and relative to the control shRenilla. Bar graphs represent mean ± SD.

D Quantification of LSD1 protein levels (top) and immunoblot of LSD1 protein levels in MKL-2 cells (bottom) normalized to loading control (H3) and relative to shRenilla.

E Individual graphs of the *in vitro* competition assay shown in Fig 1D. The three MCC cell lines WaGa, PeTa, and MKL-2 were transduced with either shLSD1.1, shLSD1.2, shRenilla, or shRPS15.

F Individual dependency plots of the three MCC cell lines PeTa, MKL-1, and MKL-2 for the genes targeted by the compound library in Fig 1A. A score of 0 indicates that a gene is not essential; correspondingly −1 is comparable to the median of all pan-essential genes. Data obtained from DepMap.

Source data are available online for this figure.

© 2020 The Authors

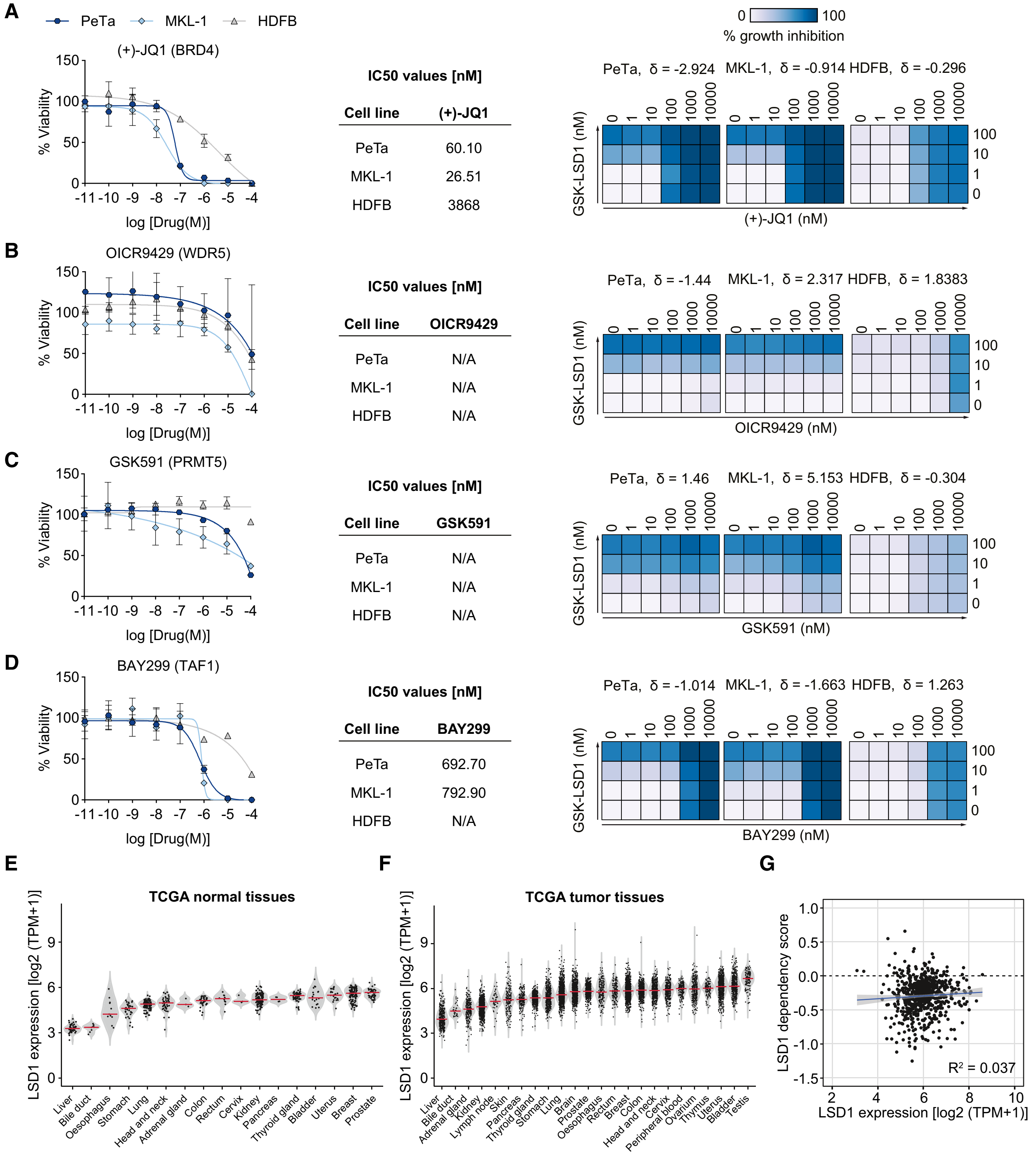

Figure EV2.

© 2020 The Authors

◀ **Figure EV2. Inhibition of the ubiquitously expressed demethylase LSD1 is unprecedented in specificity and selectivity in MCC.**

A–D Left. Dose–response curves for viability of two MCC cell lines (PeTa and MKL-1) and control HDFB cells after 6 days of treatment. $n = 4$ technical replicates. Data are represented as means ± SD. Middle. IC50 values for reduced growth of PeTa, MKL-1, and HDFB controls. Right. Bliss synergy score (δ) matrices depicting the percentage of synergistic growth inhibition in PeTa, MKL-1, and HDFB control cells upon combined treatment of GSK-LSD1 and other compounds. A positive score (δ > 0) indicates a synergistic effect, whereas a negative score (δ < 0) indicates an antagonistic effect.

E Violin plot depicting the LSD1 expression in 17 normal tissues, ordered according to mean. Red horizontal line depicts the median. Data obtained from TCGA. TPM, transcripts per million.

F Violin plot depicting the LSD1 expression in 24 tumor tissues, ordered according to mean. Red horizontal line depicts the median. Data obtained from TCGA. TPM, transcripts per million.

G Correlation plot of LSD1 expression against LSD1 dependency (RNAi screen) in various cancer types. Data obtained from DepMap RNAi and expression dataset. Blue line indicates linear regression. R^2, Pearson correlation coefficient; slope = 0.021; intercept = −0.421; $P = 0.118$; TPM, transcript per million.

Source data are available online for this figure.

© 2020 The Authors

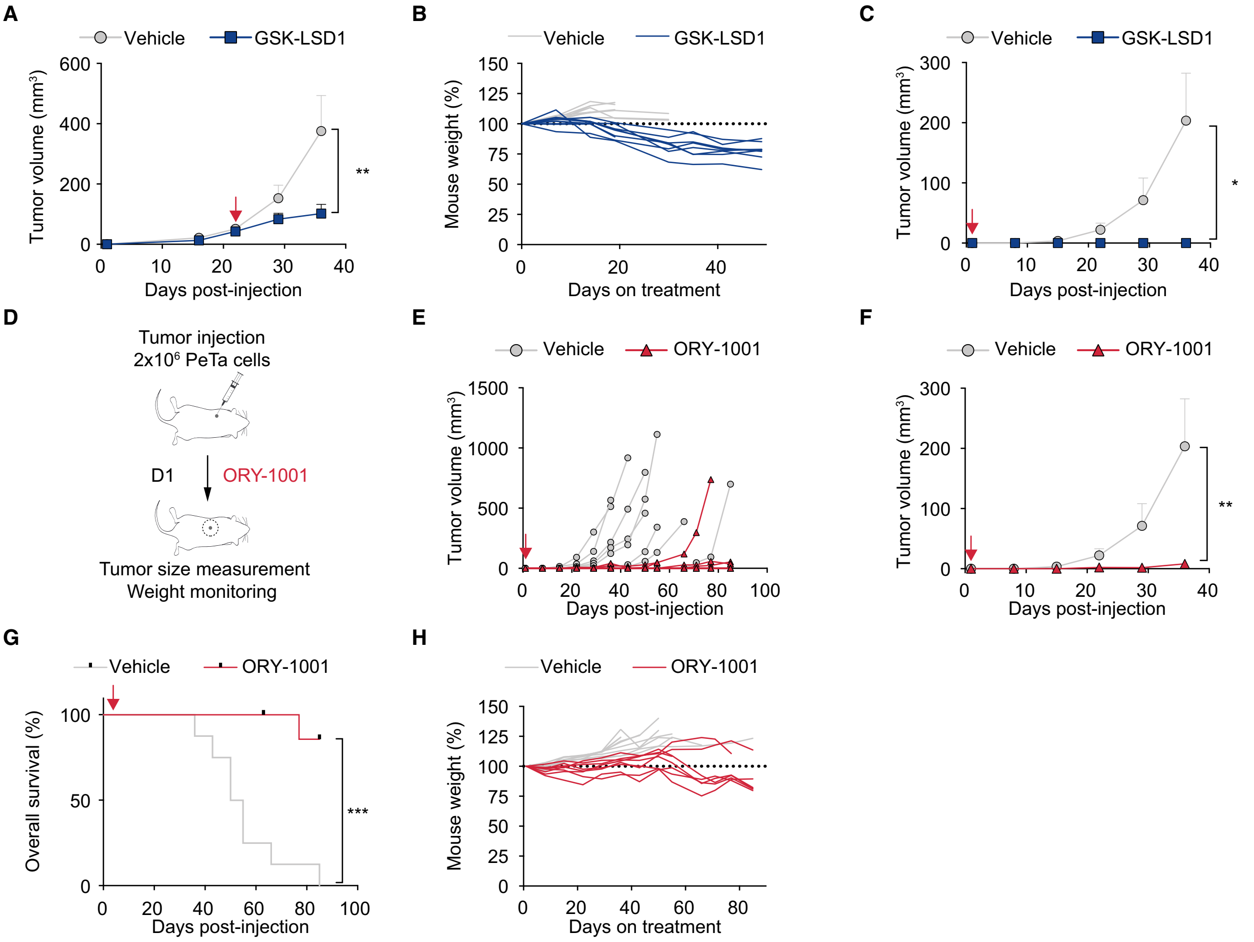

Figure EV3. Pharmacological LSD1 inhibition controls tumor growth *in vivo*.

A Treatment response of subcutaneous tumors in mice treated with GSK-LSD1 (n = 9) or vehicle (n = 8). Red arrow, start of therapy, day 22. Data are represented as means ± SEM. **P = 0.0025 (Mann–Whitney test).

B Mouse weight (%) during treatment of GSK-LSD1 (n = 9) or vehicle-treated (n = 8) mice relative to weight at treatment start (D22).

C Treatment response of subcutaneous tumors treated with GSK-LSD1 (n = 8) or vehicle (n = 8). Red arrow, start of therapy, day 1. Data are represented as means ± SEM. *P = 0.0363 (unpaired Student's t-test with Welch's correction).

D Schematic depicting the experimental setup for *in vivo* xenograft tumor treatment with ORY-1001 in NSG mice. ORY-1001 or vehicle treatment was started 1 day after tumor injection (D1).

E Individual tumor growth with ORY-1001 (n = 8) or vehicle-treated (n = 8) mice. Red arrow, start of therapy, day 1.

F Treatment response of subcutaneous tumors treated with ORY-1001 (n = 8) or vehicle (n = 8). Red arrow, start of therapy, day 1. Data are represented as means ± SEM. **P = 0.0087 (Mann–Whitney test).

G Kaplan–Meier curve of ORY-1001 (n = 8) or vehicle-treated (n = 8) mice. Mice were sacrificed when tumors reached a volume ≥ 1.5 cm^3 or greatest dimension was ≥ 1.5 cm. Red arrow, start of therapy, day 1. ***P < 0.0001 (log-rank Mantel–Cox test).

H Relative mouse weight (%) during treatment of ORY-1001 (n = 8) or vehicle-treated (n = 8) mice.

Source data are available online for this figure.

 © 2020 The Authors

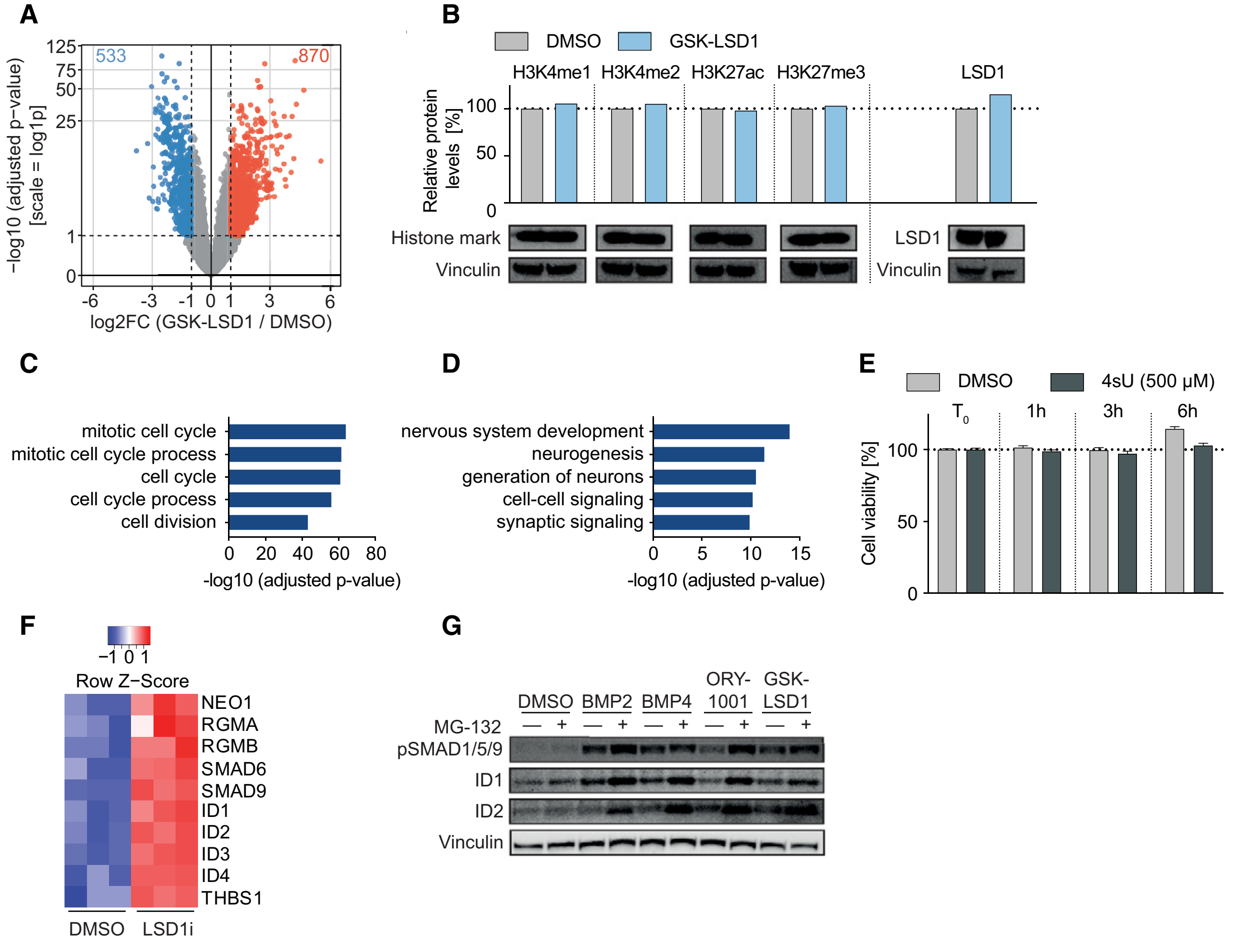

Figure EV4. LSD1 inhibition induces marked transcriptional changes.

- A Volcano plot showing the $-\log_{10}$ (adjusted *P*-value) and $\log_2$ fold change ($\log_2$FC) for transcripts detected by RNAseq analysis of PeTa cells treated with 100 nM GSK-LSD1 or DMSO for 6 days. Significantly up- and downregulated genes (FDR $\leq$ 0.05; abs[$\log_2$FC] $\geq$ 1) are marked in red and blue, respectively.
- B Immunoblot analysis for histone marks and LSD1 upon GSK-LSD1 and protein level quantification normalized to loading control (vinculin) and relative to DMSO control.
- C Pathway enrichment analysis (GO:BP) of 533 significantly downregulated genes upon GSK-LSD1 treatment displayed in Fig 4D.
- D Pathway enrichment analysis (GO:BP) of 870 significantly upregulated genes upon GSK-LSD1 treatment displayed in Fig 4F.
- E Cell viability (%) upon 4-thiouridine (4sU) treatment in PeTa cells over time relative to treatment start (T_0). Data are represented as means $\pm$ SD.
- F Heatmap depicting the transcriptional activation of members of TGFβ signaling after 24-h treatment with LSD1i.
- G Immunoblot probing for protein levels of phospho-SMAD1/5/9, ID1, and ID2 in PeTa cells treated with indicated compounds for 24 h. Vinculin serves as loading control.

Source data are available online for this figure.

Figure EV5. HMG20B is an essential LSD1-CoREST complex subunit necessary for proliferation.

A Protein–protein interaction complexes identified using Metascape analysis by LSD1 co-immunoprecipitation/mass spectrometry and adjusted *P*-values.

B Schematic and immunoblot of LSD1 co-immunoprecipitation (co-IP) in ORY-1001 (1 μM) treated PeTa cells. IB, immunoblot; MS, mass spectrometry.

C Dependency on LSD1 complex members in MCC compared to other skin cancers. Data obtained from DepMap. Median is indicated with a horizontal orange line.

D, E Violin plot depicting the dependency scores of RCOR1 (D) and GSE1 (E) in MCC compared to cancer types from 23 and 16 tissues, respectively, ordered according to mean dependency score. Red horizontal line depicts the median. Data obtained from DepMap RNAi dataset. Blood, hematopoietic and lymphoid tissue; U. aerodigestive, upper aerodigestive tract; A. ganglia, autonomic ganglia; CNS, central nervous system.

F Expression levels in CPM (counts per million) of the CoREST complex members after 24 h of 100 nM GSK-LSD1 or DMSO treatment. n = 3 biological replicates.

G Expression levels in CPM (counts per million) of the CoREST complex members after 6 days of 100 nM GSK-LSD1 or DMSO treatment. n = 3 biological replicates.

H Immunoblot of co-immunoprecipitated (co-IP) V5-tagged HMG20B domain deletion mutants. WT, wild-type; ΔALPHA, alpha-helices deletion; ΔCC, coiled-coil deletion; ΔHMG, HMG box deletion; I, input sample; E, eluted immunoprecipitated sample.

I, J Left. Dose–response curves for viability of two MCC cell lines (PeTa and MKL-1) and control HDFB cells after 6 days of treatment. n = 4 technical replicates. Data are represented as means ± SD. Middle. IC50 values for reduced growth of PeTa, MKL-1, and HDFB controls. Right. Bliss synergy score (δ) matrices depicting the percentage of synergistic growth inhibition in PeTa, MKL-1, and HDFB control cells upon combined treatment of GSK-LSD1 and other compounds. A positive score ($\delta > 0$) indicates a synergistic effect, whereas a negative score ($\delta < 0$) indicates an antagonistic effect.

Source data are available online for this figure.

 © 2020 The Authors

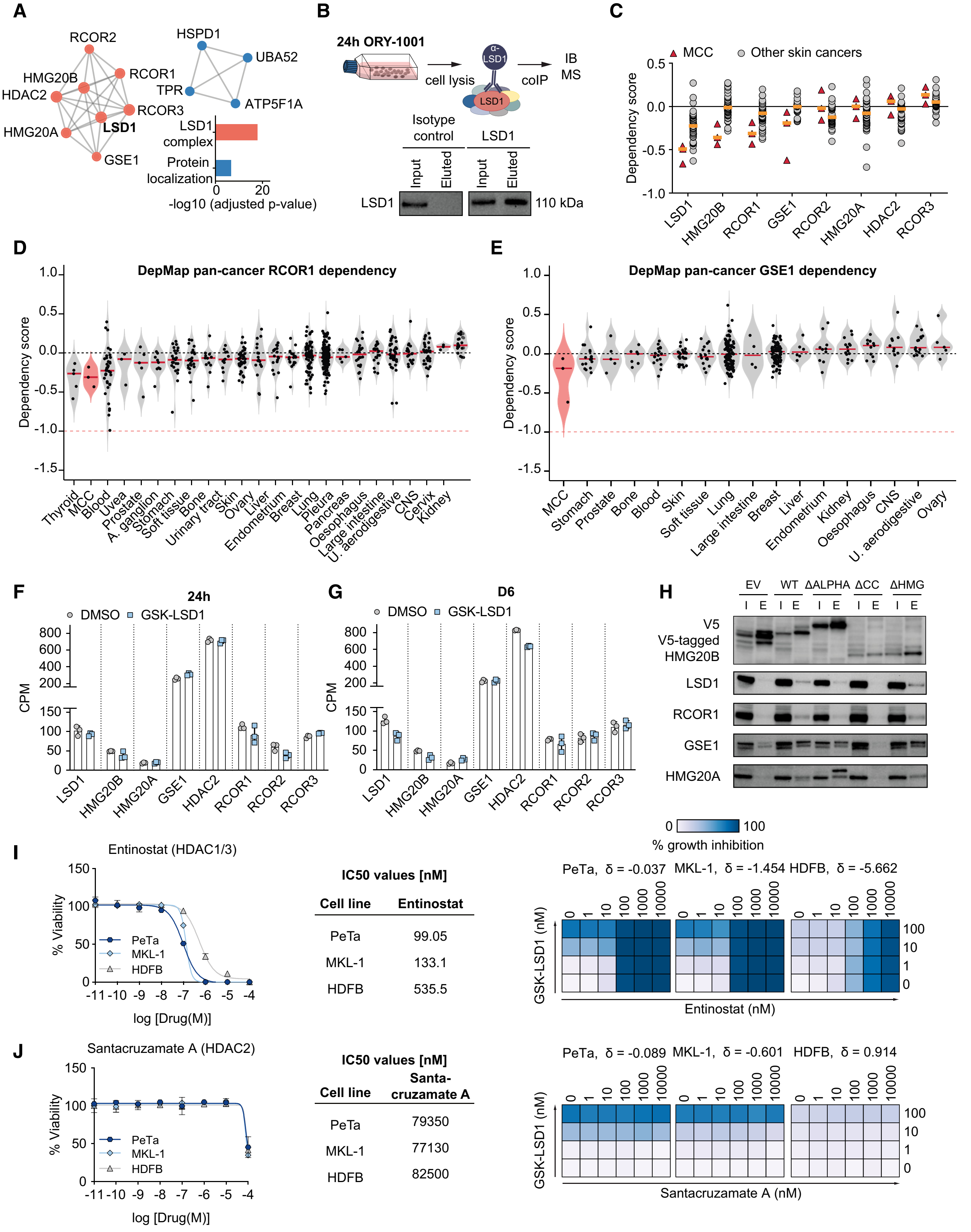
A
RCOR2
HSPD1
UBA52
HMG20B
RCOR1
HDAC2
TPR
ATP5F1A
RCOR3
HMG20A
LSD1
GSE1
LSD1 complex
Protein localization
0
20
-log10 (adjusted p-value)
B
24h ORY-1001
cell lysis
α-LSD1
LSD1
coIP
IB
MS
Isotype control
LSD1
Input
Eluted
Input
Eluted
LSD1
110 kDa
C
MCC
Other skin cancers
Dependency score
0.5
0.0
-0.5
-1.0
LSD1
HMG20B
RCOR1
GSE1
RCOR2
HMG20A
HDAC2
RCOR3
D
DepMap pan-cancer RCOR1 dependency
Dependency score
1.0
0.5
0.0
-0.5
-1.0
-1.5
Thyroid
MCC
Blood
Uvea
Prostate
A. ganglion
Stomach
Soft tissue
Bone
Urinary tract
Skin
Ovary
Liver
Endometrium
Breast
Lung
Pleura
Pancreas
Oesophagus
Large intestine
U. aerodigestive
CNS
Cervix
Kidney
E
DepMap pan-cancer GSE1 dependency
Dependency score
1.0
0.5
0.0
-0.5
-1.0
-1.5
MCC
Stomach
Prostate
Bone
Blood
Skin
Soft tissue
Lung
Large intestine
Breast
Liver
Endometrium
Kidney
Oesophagus
CNS
U. aerodigestive
Ovary
F
24h
DMSO
GSK-LSD1
CPM
800
600
400
200
150
100
50
0
LSD1
HMG20B
HMG20A
GSE1
HDAC2
RCOR1
RCOR2
RCOR3
G
D6
DMSO
GSK-LSD1
CPM
800
600
400
200
150
100
50
0
LSD1
HMG20B
HMG20A
GSE1
HDAC2
RCOR1
RCOR2
RCOR3
H
EV
WT
ΔALPHA
ΔCC
ΔHMG
I E I E I E I E I E
V5
V5-tagged HMG20B
LSD1
RCOR1
GSE1
HMG20A
0
100
% growth inhibition
I
Entinostat (HDAC1/3)
% Viability
150
100
50
0
PeTa
MKL-1
HDFB
-11 -10 -9 -8 -7 -6 -5 -4
log [Drug(M)]
IC50 values [nM]
Cell line
Entinostat
PeTa
99.05
MKL-1
133.1
HDFB
535.5
PeTa, δ = -0.037
MKL-1, δ = -1.454
HDFB, δ = -5.662
0 1 10 100 1000 10000
GSK-LSD1 (nM)
100
10
1
0
Entinostat (nM)
J
Santacruzamate A (HDAC2)
% Viability
150
100
50
0
PeTa
MKL-1
HDFB
-11 -10 -9 -8 -7 -6 -5 -4
log [Drug(M)]
IC50 values [nM]
Cell line
Santa-cruzamate A
PeTa
79350
MKL-1
77130
HDFB
82500
PeTa, δ = -0.089
MKL-1, δ = -0.601
HDFB, δ = 0.914
0 1 10 100 1000 10000
GSK-LSD1 (nM)
100
10
1
0
Santacruzamate A (nM)

Figure EV5.
