# Supplementary figures and images for: LSD1 inhibition induces differentiation and cell death in Merkel cell carcinoma
Source: EMBO Mol Med. 2020 Oct 7;12(11):e12525. doi: 10.15252/emmm.202012525 (PMC7645387; doi:10.15252/emmm.202012525)

Figure 3

D

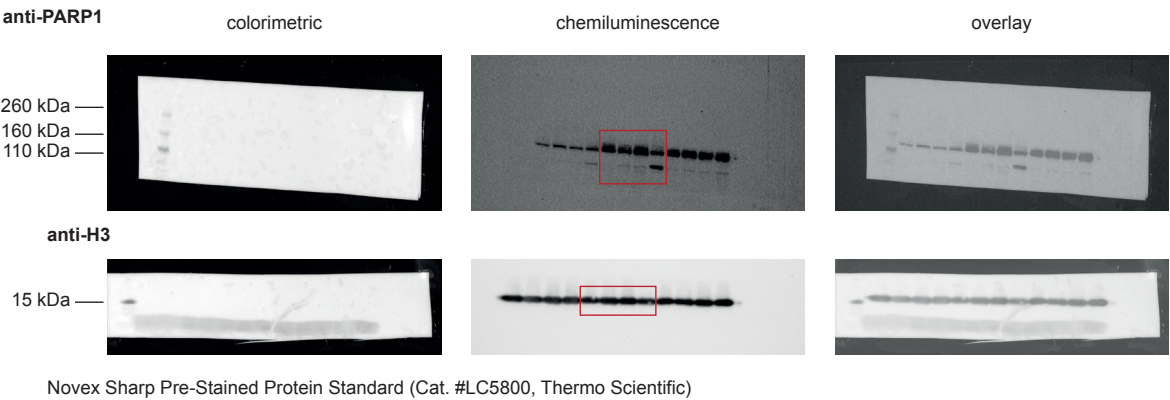

Supplement: Supplementary file 7 — Source Data for Figure 3 [file EMMM-12-e12525-s005.zip › EMM-2020-12525_SourceDataforFigure3/EMM-2020-12525_SourceDataforFigure3.pdf]

**Figure 6**

**A**

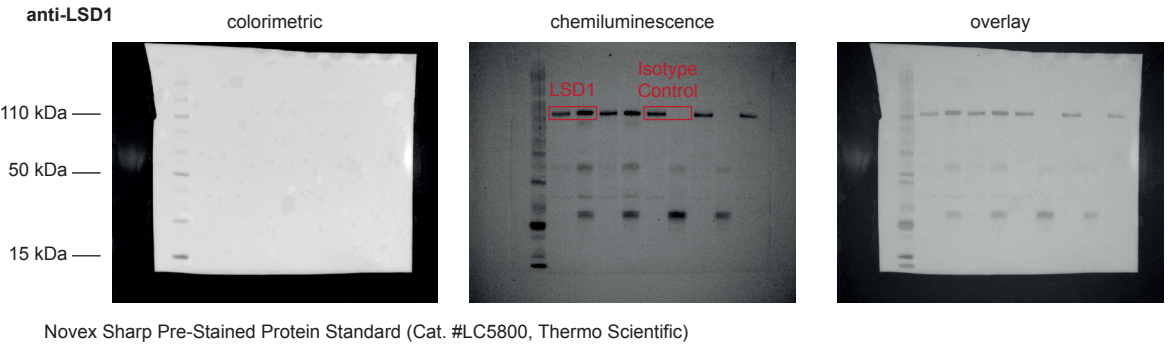

**H**

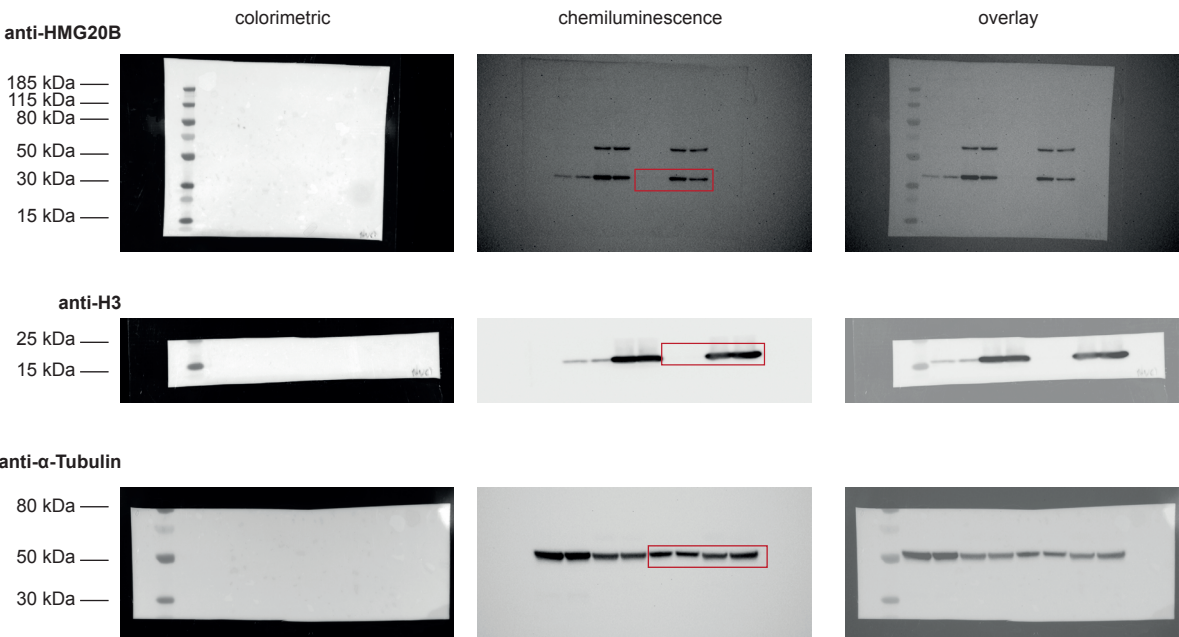

Supplement: Supplementary file 10 — Source Data for Figure 6 [file EMMM-12-e12525-s008.zip › EMM-2020-12525_SourceDataforFigure6/EMM-2020-12525_SourceDataforFigure6.pdf]
