# Supplementary material for: LSD1 inhibition induces differentiation and cell death in Merkel cell carcinoma
Source: EMBO Mol Med. 2020 Oct 7;12(11):e12525. doi: 10.15252/emmm.202012525 (PMC7645387; doi:10.15252/emmm.202012525)
Supplement: Supplementary file 11 — Source Data for Figure 7 [file EMMM-12-e12525-s009.zip › EMM-2020-12525_SourceDataforFigure7/EMM-2020-12525_SourceDataforFigure7.pdf]

Figure 7

C

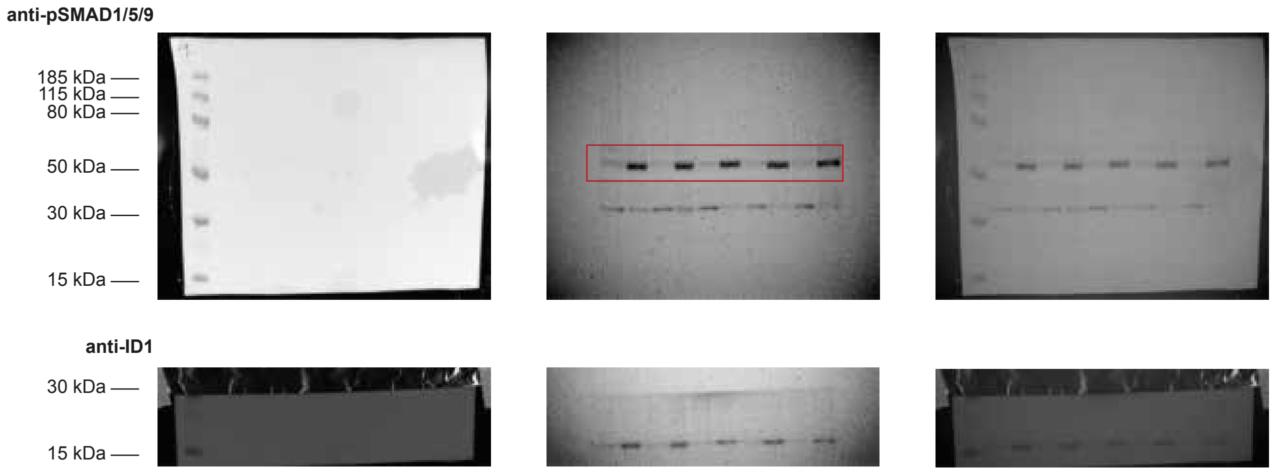

Remark: The upper part of the blot was cover while imaging to cover the strong signal from the previous antibody.

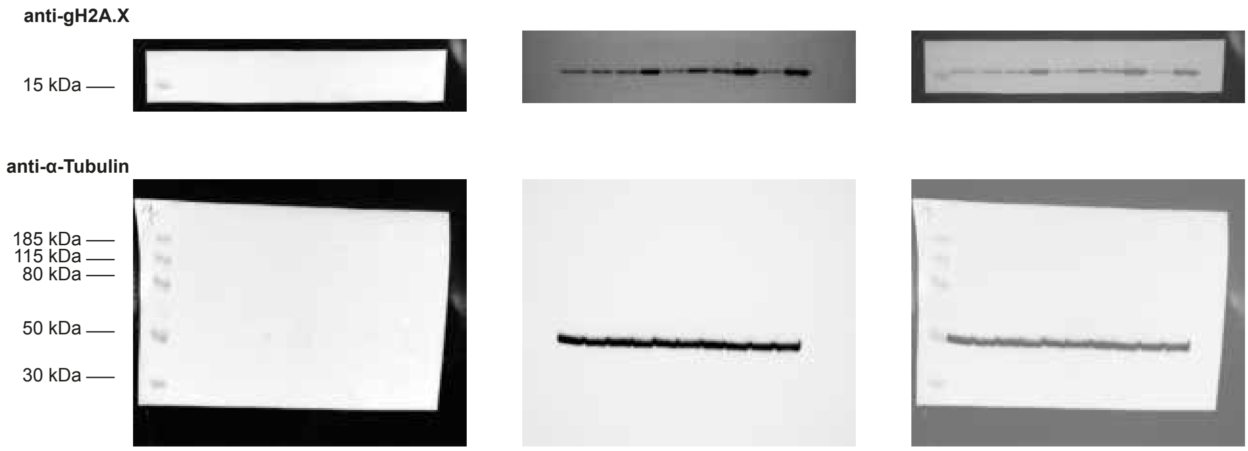

Page Ruler Plus Prestained Protein Ladder (Cat. #26619, Thermo Scientific)

Figure 7

D

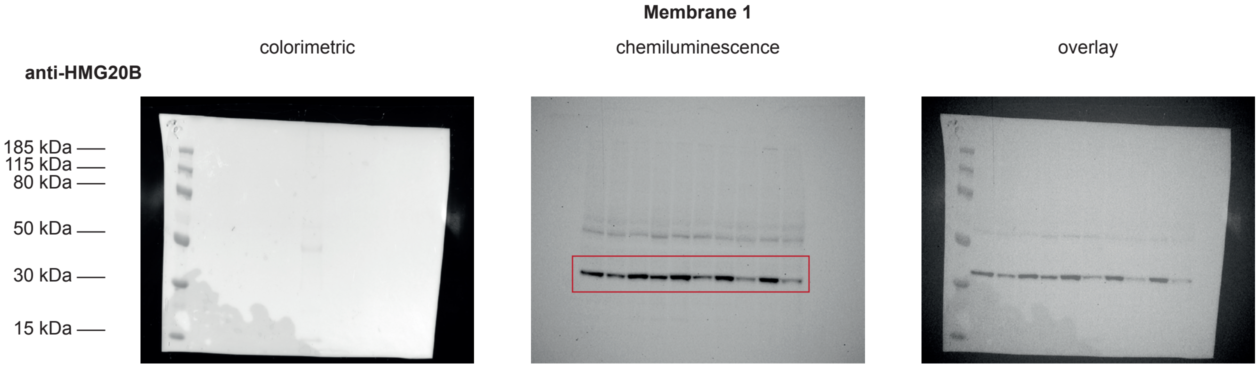

Remark: loading control is in Figure 7C (same membrane).

Page Ruler Plus Prestained Protein Ladder (Cat. #26619, Thermo Scientific)

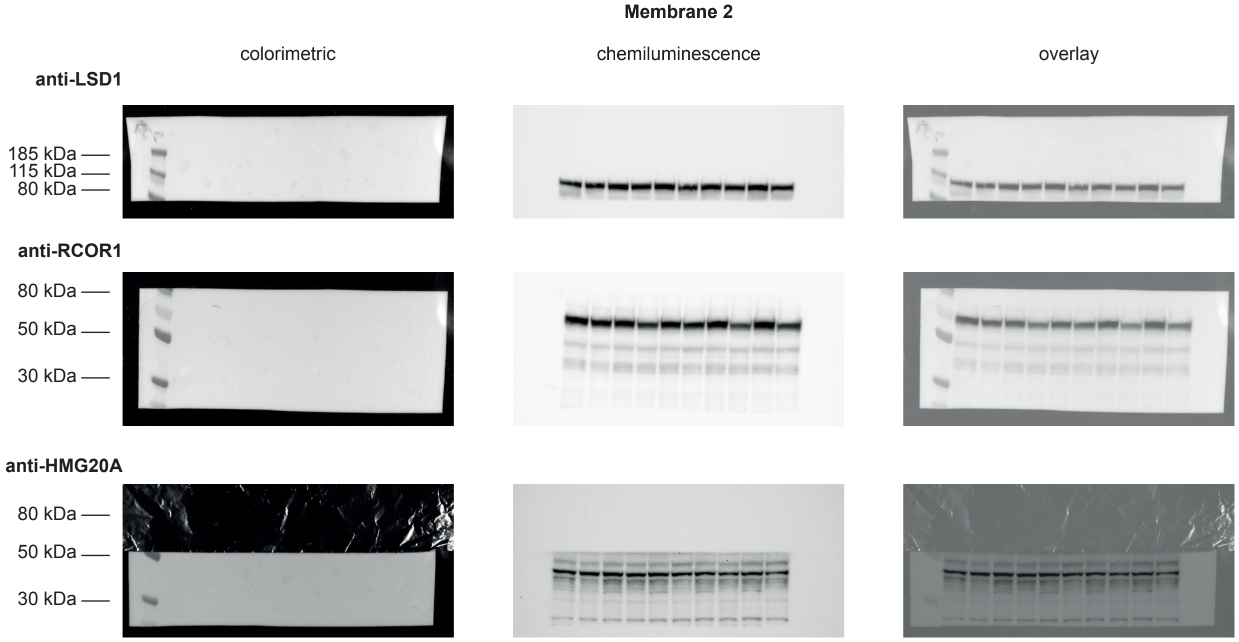

Remark: The upper part of the blot was cover while imaging to cover the strong signal from the previous antibody.

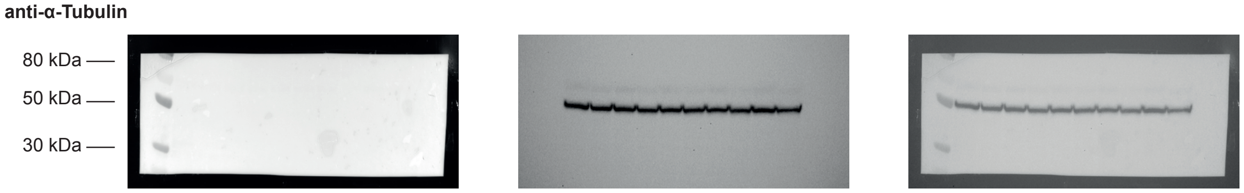

Page Ruler Plus Prestained Protein Ladder (Cat. #26619, Thermo Scientific)
