# Supplementary material for: LSD1 inhibition induces differentiation and cell death in Merkel cell carcinoma
Source: EMBO Mol Med. 2020 Oct 7;12(11):e12525. doi: 10.15252/emmm.202012525 (PMC7645387; doi:10.15252/emmm.202012525)
Supplement: Supplementary file 3 — Source Data for Expanded View [file EMMM-12-e12525-s010.zip › EMM-2020-12525_SourceDataforExpandedViewFigure4.zip!/EMM-2020-12525_SourceDataforExpandedViewFigure4/EMM-2020-12525_SourceDataforFigureEV4.pdf]

Figure EV4

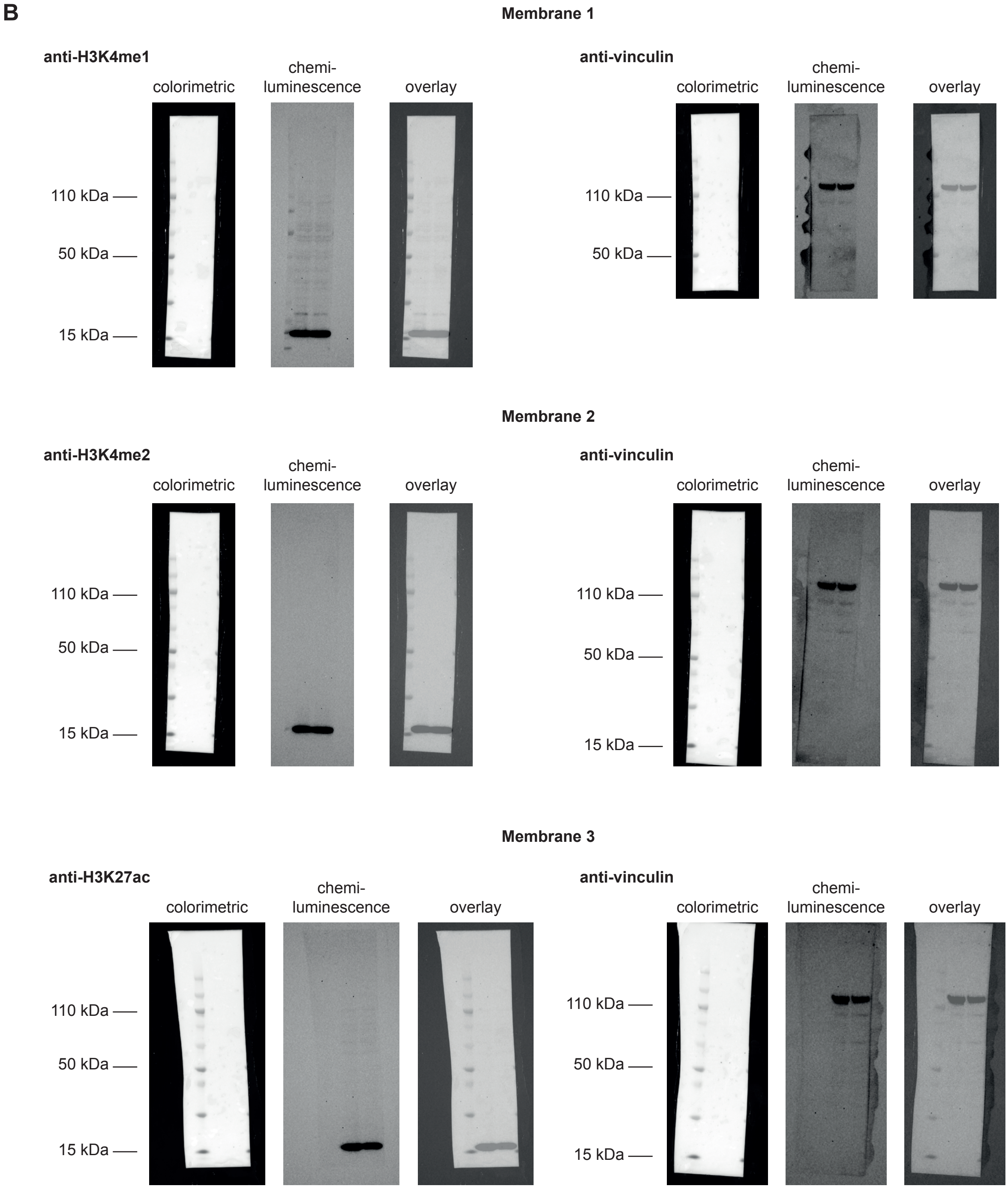
B
Membrane 1
anti-H3K4me1
colorimetric
chemi-luminescence
overlay
110 kDa
50 kDa
15 kDa
anti-vinculin
colorimetric
chemi-luminescence
overlay
110 kDa
50 kDa
Membrane 2
anti-H3K4me2
colorimetric
chemi-luminescence
overlay
110 kDa
50 kDa
15 kDa
anti-vinculin
colorimetric
chemi-luminescence
overlay
110 kDa
50 kDa
15 kDa
Membrane 3
anti-H3K27ac
colorimetric
chemi-luminescence
overlay
110 kDa
50 kDa
15 kDa
anti-vinculin
colorimetric
chemi-luminescence
overlay
110 kDa
50 kDa
15 kDa

Figure EV4

B

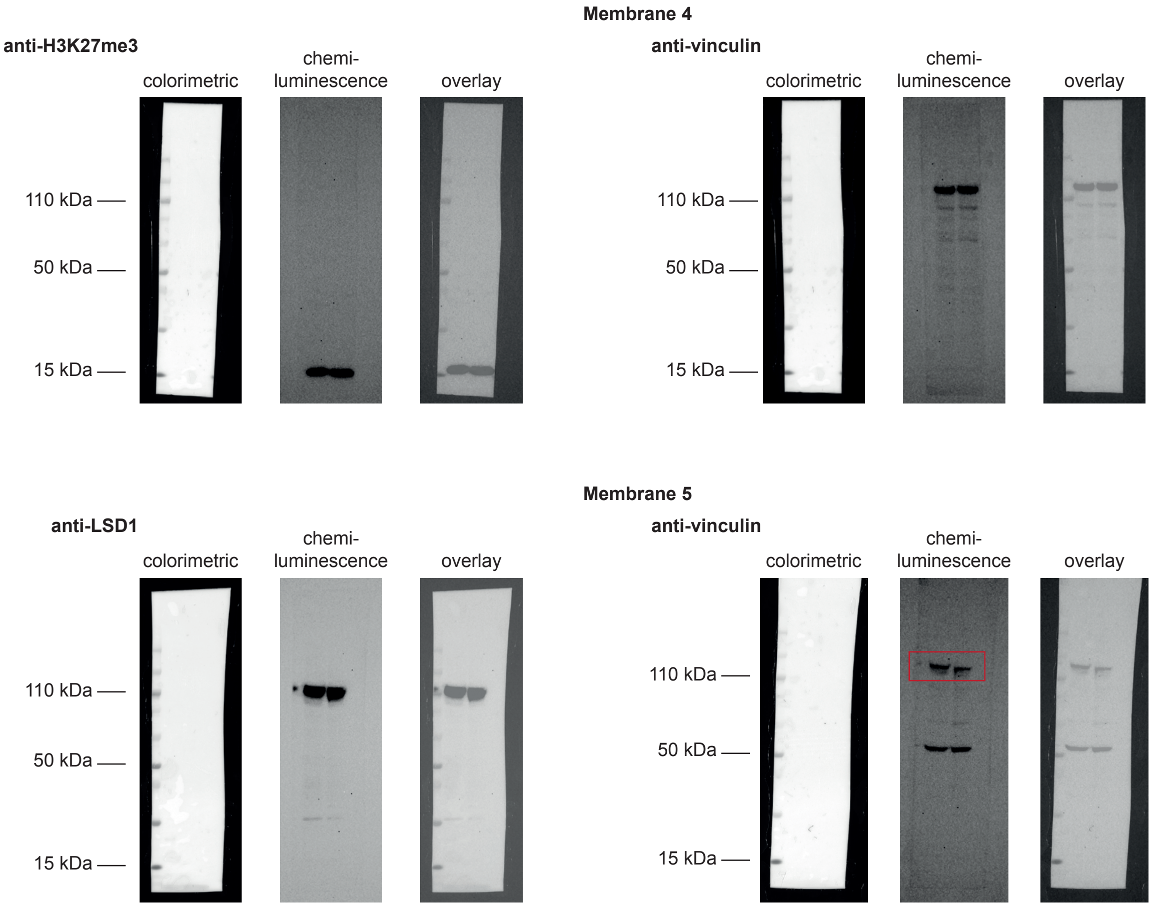

Novex Sharp Pre-Stained Protein Standard (Cat. #LC5800, Thermo Scientific)

Figure EV4

G

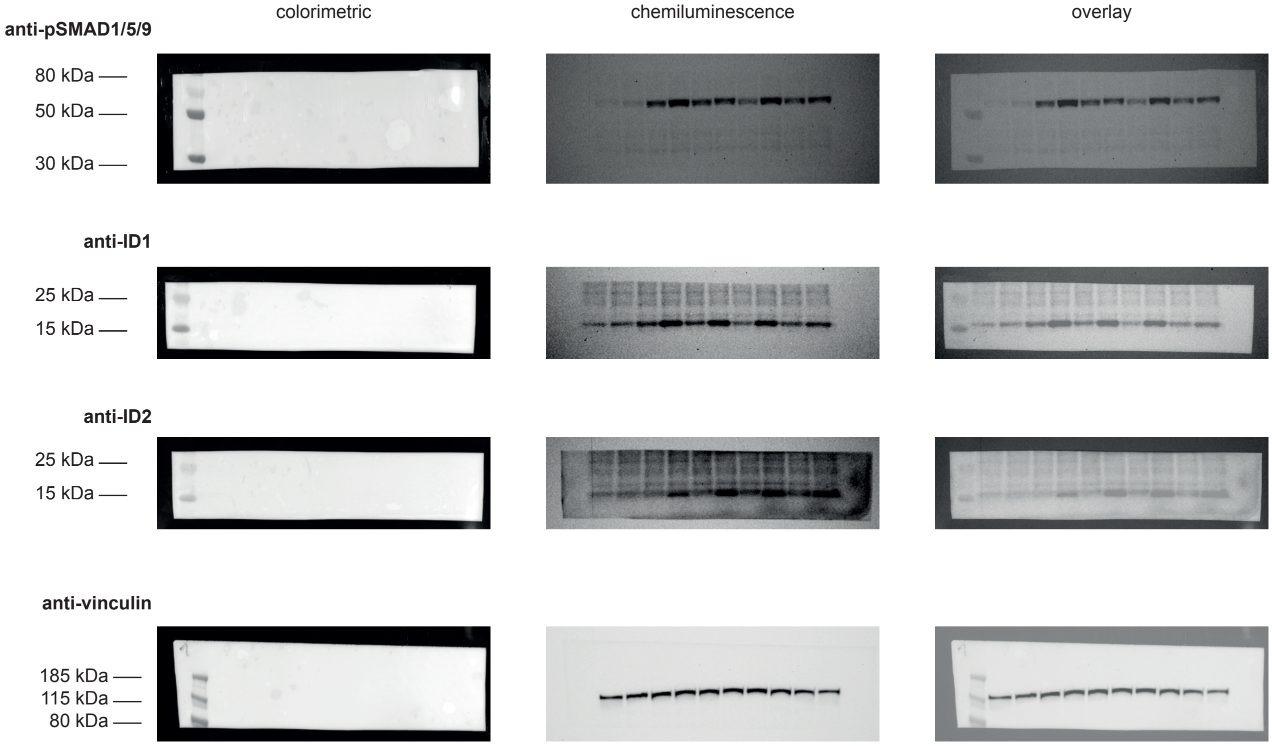

Page Ruler Plus Prestained Protein Ladder (Cat. #26619, Thermo Scientific)
