# Supplementary material for: LSD1 inhibition induces differentiation and cell death in Merkel cell carcinoma
Source: EMBO Mol Med. 2020 Oct 7;12(11):e12525. doi: 10.15252/emmm.202012525 (PMC7645387; doi:10.15252/emmm.202012525)
Supplement: Supplementary file 3 — Source Data for Expanded View [file EMMM-12-e12525-s010.zip › EMM-2020-12525_SourceDataforExpandedViewFigure5.zip!/EMM-2020-12525_SourceDataforExpandedViewFigure5/EMM-2020-12525_SourceDataforFigureEV5.pdf]

Figure EV5

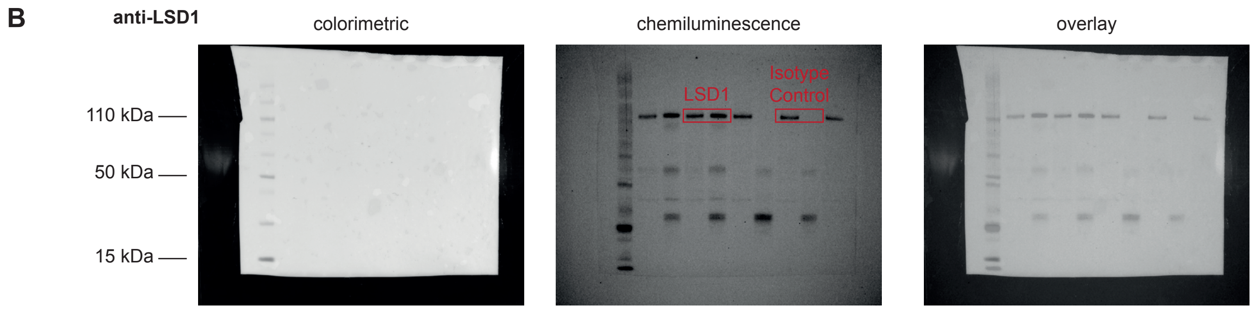

Novex Sharp Pre-Stained Protein Standard (Cat. #LC5800, Thermo Scientific)

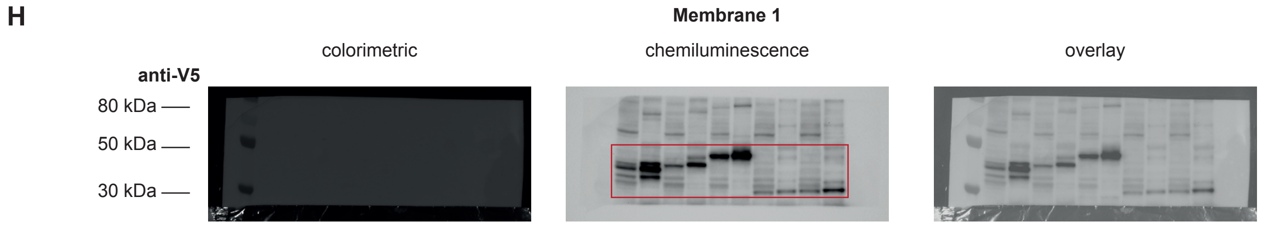

Remark: The lower part of the blot was cover while imaging to cover the strong signal from the heavy chain of the coIP antibody.

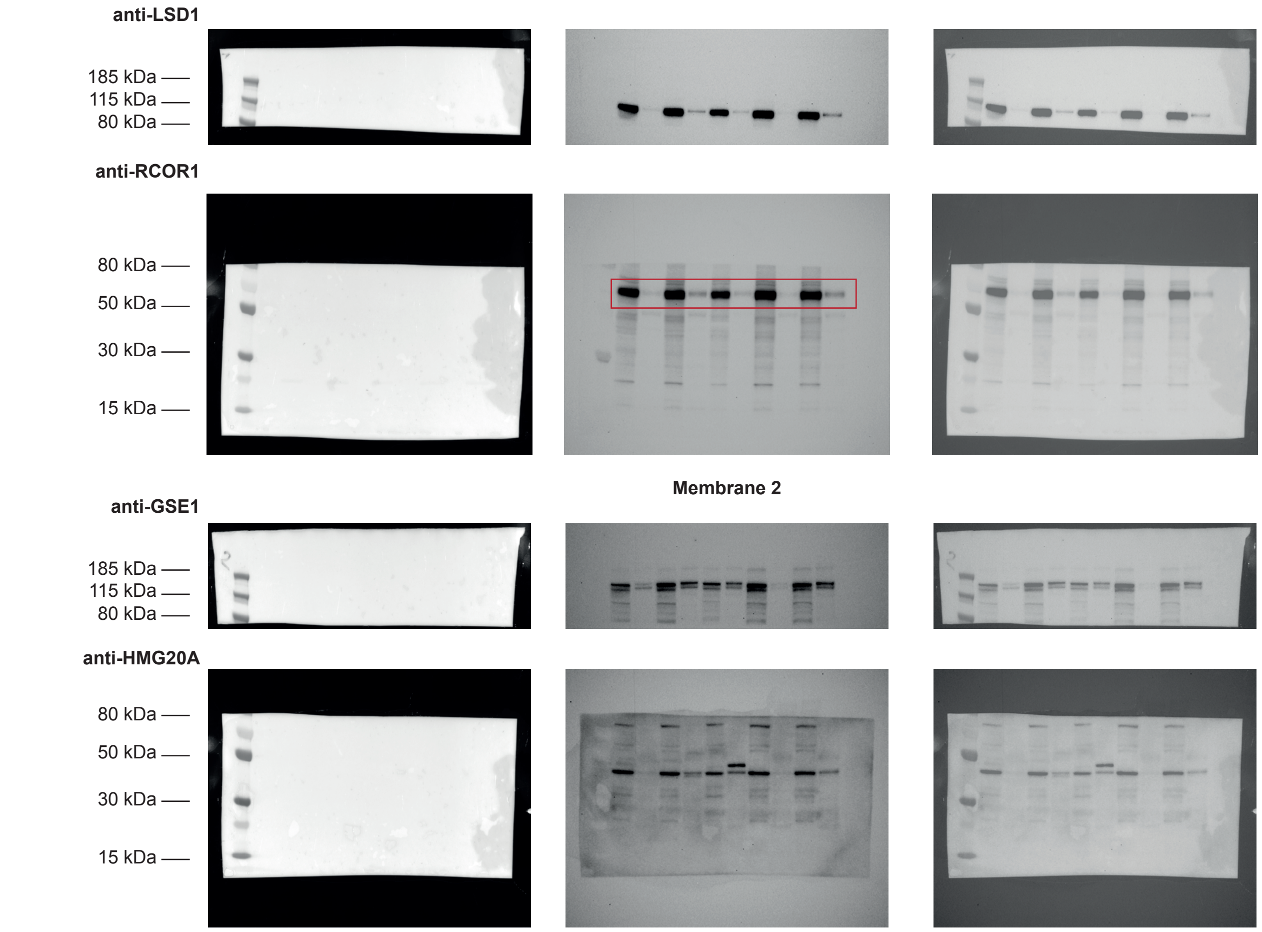

Page Ruler Plus Prestained Protein Ladder (Cat. #26619, Thermo Scientific)
