# Supplementary material for: LSD1 inhibition induces differentiation and cell death in Merkel cell carcinoma
Source: EMBO Mol Med. 2020 Oct 7;12(11):e12525. doi: 10.15252/emmm.202012525 (PMC7645387; doi:10.15252/emmm.202012525)
Supplement: Supplementary file 3 — Source Data for Expanded View [file EMMM-12-e12525-s010.zip › EMM-2020-12525_SourceDataforExpandedViewFigure1.zip!/EMM-2020-12525_SourceDataforExpandedViewFigure1/EMM-2020-12525_SourceDataforFigureEV1.pdf]

Figure EV1

D

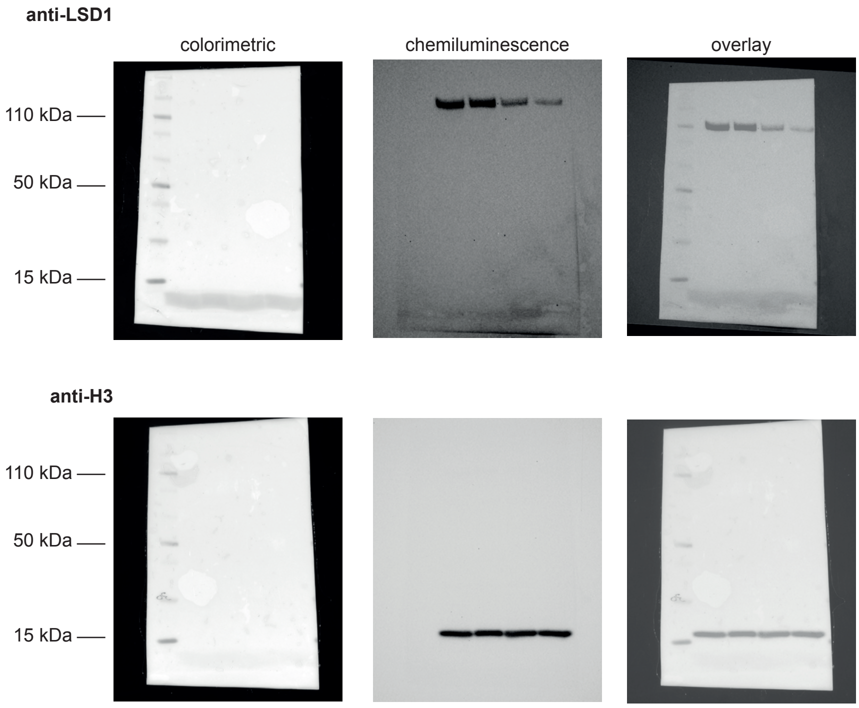

Novex Sharp Pre-Stained Protein Standard (Cat. #LC5800, Thermo Scientific)
